# Academic practice in ecology and evolution: Soliciting a new category of manuscript

**DOI:** 10.1002/ece3.3200

**Published:** 2017-06-28

**Authors:** Allen J. Moore, Jennifer Firn, Andrew Beckerman

**Affiliations:** ^1^ Department of Entomology University of Georgia Athens GA USA; ^2^ School of Earth, Environmental & Biological Sciences Queensland University of Technology Brisbane QLD Australia; ^3^ Plant and Animal Sciences University of Sheffield Sheffield UK

We are excited to announce a new category of paper: “Academic Practice in Ecology and Evolution.”

As ecologists and evolutionary biologists, we apply scholarly approaches to the myriad roles we have undertaken in our professions. Publishing about such new knowledge and advances in our ‘roles’ (e.g., teaching, service, outreach, professional development, and change) typically occurs in a range of transdisciplinary journals. Tracking down this literature, in what can be disparate fields of research, is time‐consuming and can prevent groundbreaking ideas from being more generally acknowledged and ultimately implemented in the day‐to‐day.

Our new category “Academic Practice” is intended to remedy this situation and bring high‐quality studies in the following broad categories to the attention of our readers:

- advancements and critical evaluations of teaching pedagogies, curriculum, and assessment for student learning
- how our fields respond to publication trends and bias (e.g., authorship, gender, blind review, and replication)
- critical analyses of student learning and perception of the efficacies of pedagogies, curriculum, and assessment
- student‐led capstone/final‐year projects that include rigorous evaluations of learning and teaching around these experiences
- studies focused on integrated digital technologies, work‐integrated learning, and other novel approaches to student learning.

We intend that “Academic Practice” be broadly interpreted, and welcome presubmission questions if you have a study that does not fall neatly into these categories. We have already begun to receive papers that are more about our profession than our scientific results (e.g., Fox & Burns, 2015), and we see this as an increasingly important activity in our field. As a journal that seeks to innovate, we therefore hope to provide an outlet for such novel studies.

We will evaluate submissions under “Academic Practice” in light of our perception of their value to ecologists and evolutionary biologists as well as the rigor of analysis. No matter the topic, we expect studies to have objective evaluation. While such studies may be more social science than life science, we expect that the value for practicing researchers and Academics will be made obvious.

Increasingly, researchers are integrating their roles as educators into their funded projects. Of course, we have always trained personnel during our research, by supporting technical help, postgraduates and postdoctoral scholars, but there is a positive movement toward developing teaching tools, investigating student learning, and documenting and evaluating policy effects. Grant applications often have a requirement for explicit value added (“Broader Impacts” section at NSF in the United States; “Pathways to Impact” for NERC in the UK). Proposing interesting or exciting ideas for outreach, learning tools, and activities are fine, but granting agencies also want the researchers to evaluate effectiveness and for peer review. Thus, like the proposed research itself, they expect outputs that are peer reviewed. There needs to be outlets for these outputs, and a trusted and managed peer review. Granting agencies that recognize that these outputs may incur publication costs will budget for these activities. There are, of course, other outlets for manuscripts that report studies investigating biology education research. Biology education research is a specific professional field of study, and is particularly strong, and at least in the United States is very well‐funded by the National Science Foundation. The NSF has the Directorate for Education and Human Resources—EHR—that supports “STEM education at all levels” and has divisions within the directorate of Division of Research on Learning in Formal and Informal Settings (DRL) and the Division of Undergraduate Education (DUE) that fund projects seeking to evaluate and improve student learning in STEM fields. We do not envision competing with the traditional outlets for such work; however, such journals are typically directed toward the professionals in the field of biology education research (e.g., *CBE—Life Sciences Education*). They only occasionally focus on specialized areas of biology such as ecology and evolution.

We hope, against this backdrop, to provide an outlet for papers that are directed toward practicing scientists in ecology and evolution. The work will be peer reviewed and assessed for potential value to practicing researchers and scholars. As always, we look to the community to define the scope of this activity. Creativity and an interest in students and training have always defined our fields, and we hope to facilitate this further.
